# Two Distinct Isoforms of Matrix Metalloproteinase-2 Are Associated with Human Delayed Kidney Graft Function

**DOI:** 10.1371/journal.pone.0136276

**Published:** 2015-09-17

**Authors:** Shaynah Wanga, Carla S. Ceron, Cynthia Delgado, Sunil K. Joshi, Kimberly Spaulding, Joy P. Walker, Sangheon Song, Jean L. Olson, David H. Lovett

**Affiliations:** 1 Department of Medicine, San Francisco Department of Veterans Affairs Medical Center/University of California San Francisco, San Francisco, California, United States of America; 2 Department of Surgery, San Francisco Department of Veterans Affairs Medical Center/University of California San Francisco, San Francisco, California, United States of America; 3 Department of Pathology, University of California San Francisco, San Francisco, California, United States of America; Center for Molecular Biotechnology, ITALY

## Abstract

Delayed graft function (DGF) is a frequent complication of renal transplantation, particularly in the setting of transplantation of kidneys derived from deceased donors and expanded-criteria donors. DGF results from tubular epithelial cell injury and has immediate and long term consequences. These include requirement for post-transplantation dialysis, increased incidence of acute rejection, and poorer long-term outcomes. DGF represents one of the clearest clinical examples of renal acute ischemia/reperfusion injury. Experimental studies have demonstrated that ischemia/reperfusion injury induces the synthesis of the full length secreted isoform of matrix metalloproteinase-2 (FL-MMP-2), as well as an intracellular N-terminal truncated MMP-2 isoform (NTT-MMP-2) that initiates an innate immune response. We hypothesized that the two MMP-2 isoforms mediate tubular epithelial cell injury in DGF. Archival renal biopsy sections from 10 protocol biopsy controls and 41 cases with a clinical diagnosis of DGF were analyzed for the extent of tubular injury, expression of the FL-MMP-2 and NTT-MMP-2 isoforms by immunohistochemistry (IHC), in situ hybridization, and qPCR to determine isoform abundance. Differences in transcript abundance were related to tubular injury score. Markers of MMP-2-mediated injury included TUNEL staining and assessment of peritubular capillary density. There was a clear relationship between tubular epithelial cell expression of both FL-MMP-2 and NTT-MMP-2 IHC with the extent of tubular injury. The MMP-2 isoforms were detected in the same tubular segments and were present at sites of tubular injury. qPCR demonstrated highly significant increases in both the FL-MMP-2 and NTT-MMP-2 transcripts. Statistical analysis revealed highly significant associations between FL-MMP-2 and NTT-MMP-2 transcript abundance and the extent of tubular injury, with NTT-MMP-2 having the strongest association. We conclude that two distinct MMP-2 isoforms are associated with tubular injury in DGF and offer novel therapeutic targets for the prevention of this disorder.

## Introduction

Delayed graft function (DGF) is a frequent complication of renal transplantation and has been attributed to the effects of acute ischemia/reperfusion injury [1]. DGF is an operational diagnosis and generally denotes a requirement for at least one episode of dialysis within the first week following transplantation [2]. DGF occurs in approximately 24 percent of kidney transplants according to The Organ Procurement and Transplantation Network, and is more common in cadaveric kidney transplants, in particular renal transplants from cardiac death donors and expanded-criteria donors (ECD) [3]. Due to increasing use of kidneys obtained from deceased and ECD donors, one may anticipate a future increase in DGF rates. Recent studies have shown that DGF has adverse effects on both short and long term outcomes of transplant function. However, the mechanisms involved remain incompletely defined [4–6].

Renal ischemia/reperfusion injury occurs at the time when the non-perfused donor kidney is connected to the recipient circulation. This event triggers a rapid and large release of molecules that contribute to oxidant stress injury, including reactive oxygen species and peroxynitrites [7,8]. These highly reactive molecules directly affect the structure and function of cellular protein and lipid components via denaturation. In addition, reactive oxygen species activate multiple signaling cascades, including the AP-1, NF-κB and NFAT transcription networks, that further contribute to injury [9–12]. Experimental studies of acute ischemia/reperfusion injury in multiple organs, including the kidney, have emphasized a critical role for matrix metalloproteinases in the evolution of organ injury. One discrete matrix metalloproteinase, matrix metalloproteinase-2 (MMP-2) has been the focus of considerable attention in rodent models of ischemia/reperfusion injury [13,14]. Murine models of ischemia/reperfusion injury have demonstrated that the secreted form of MMP-2 is rapidly induced in tubular epithelial cells and that non-selective MMP inhibition or use of MMP-2 knockout mice reduces the extent of tubular epithelial cell injury [14,15]. Previous studies from our laboratory have defined the transcriptional regulatory mechanisms that drive rapid synthesis of secreted MMP-2 [16–18]. Further, we demonstrated, within the context of transgenic renal proximal tubule expression of MMP-2, that expression of this gene was sufficient to recapitulate all of the features of progressive human renal disease, including loss of nephron mass, interstitial fibrosis, and inflammation [19].

Until recently, studies on the pathophysiologic roles of MMP-2 in renal disease have focused on the secreted form of MMP-2 (denoted in this report as full length MMP-2, FL-MMP-2), with an emphasis on modulation of the extracellular matrix and tubular basement membrane [13, 14,19]. We recently identified a novel intracellular isoform of MMP-2 generated by redox-stress mediated activation of a latent promoter located within the first intron of the MMP-2 gene. This generates a protein beginning at M^77^ that lacks the secretory signal sequence and the inhibitory prodomain [20]. The **N**-**t**erminal **t**runcated MMP-2 isoform (denoted NTT-MMP-2) is intracellular, enzymatically active, and is localized, in part, in the intramembranous space of the mitochondria. The NTT-MMP-2 isoform triggers mitochondrial-to- nuclear stress signaling with activation of NF-κB and NFAT transcriptional cascades. We reported that NTT-MMP-2 activates a primary innate immune response with enhanced transcription of genes characteristic of a Type I interferon response [20]. These include genes associated with inflammatory cell infiltration and cell death. More recent efforts have demonstrated that renal tubular epithelial cell specific transgenic expression of the NTT-MMP-2 isoform induces regulated necrosis and activation of an innate immune response, S1 Fig.

The experimental studies outlined above strongly suggested that analysis of the FL-MMP-2 and NTT-MMP-2 isoforms within the setting of human renal acute ischemia/reperfusion injury, as modeled by DGF, could potentially provide important new insights into the pathogenesis of the more common forms of acute kidney injury occurring as a consequence of cardiac surgery or contrast nephropathy.

The specific aim of the current study was to determine the association of FL-MMP-2 and NTT-MMP-2 expression with renal transplant delayed graft function as a defined clinical model of acute renal ischemia/reperfusion injury. We hypothesized that the FL-MMP-2 and NTT-MMP-2 isoforms are induced by graft ischemia/reperfusion injury and that the degree of FL-MMP-2 and NTT-MMP-2 expression would be associated with the severity of tubular epithelial cell injury. This report details the confirmation of our initial working hypothesis concerning the pathophysiologic roles of MMP-2 in this setting and demonstrates a strong association between both FL-MMP-2 and NTT-MMP-2 expression and tubular injury.

## Materials and Methods

### Clinical data and renal biopsy sources

This study was approved by the UCSF/SFVAMC Human Research Protection Program, Committee on Human Research. The study was entitled “Expression of an N-terminal truncated matrix metalloproteinase-2 isoform in human renal disease” (IRBG# 12–08789). Informed consent was waived as patient records and information were anonymized and de-identified prior to analysis.

We used the existing clinical database from the UC San Francisco (UCSF) Renal Transplant Program linked with archived clinically indicated renal biopsies. Case selection was restricted to adults over the age of 18 who received a renal transplant at UCSF during the period of 2009 to 2012 and in whom both clinical data and biopsy samples were available. The selection criterion of cases was based on the clinical diagnosis of delayed kidney graft function without histopathologic evidence of rejection by standard Banff criteria. Control biopsies were selected from the same source population and were comprised of protocol biopsies performed on patients with normal transplant function. Clinical characteristics on all cases were abstracted from the electronic medical record by a team member not involved in the laboratory procedures. The clinical data collected included recipient age, gender, dialysis vintage, etiology of kidney disease, cold ischemia time, warm ischemia time, and the donor source. Investigational approval was received by the Committee on Human Research at UCSF.

### Case adjudication

The hematoxylin and eosin stained slides from the original biopsies were reviewed by a renal pathologist without knowledge of the experimental group or the diagnoses rendered at the time of biopsy. The entire biopsy core was examined to determine the injury score. The slides were evaluated using an a priori classification scheme for tubular injury (Table 1). The tubular injury score assigned was the outcome of interest. After classification, biopsy cases were stained for both isoforms of MMP-2, FL and NTT, by laboratory staff blinded to the diagnosis of DGF. These stains were scored by the renal pathologist blinded to clinical data using the criteria outlined in Table 2.

**Table 1:** Characteristics of Study Population by Transplant Type.

	All N = 51	Deceased N = 40	Living Related N = 3	Living N = 8	P-Value
**Male**	72.5%	72.5%	100%	62.5%	0.46
**Mean Age, yrs [sd]**	54 [11.9]	55.6 [11.1]	53 [17.6]	46 [12.3]	0.60
**Race/ethnicity**					0.17
** White**	25%	30%	33%	0%	
** Black**	21%	22%	0%	25%	
** Asian**	27%	30%	0%	25%	
** Hispanic**	23%	22%	66%	12%	0.16
***Clinical Characteristics***					
** Cardiac Disease**	33%	35%	33%	25%	0.95
** Hypertension**	92%	92%	100%	87%	0.77
** Diabetes**	49%	57%	33%	25%	0.06
** Dialysis Vintage, months [std]**	54[46]	58 [40]	4.5[6.3]	47 [72]	0.045
***Etiology of Renal Disease***					
** Diabetes**	33%	40%	0%	1%	
** Hypertension**	31%	32%	33%	37%	
** Other**	33%	27%	33%	50%	
***Transplant Characteristics***					
** Cold Ischemia Time [hours]**	8.47 [7.13]	9.7 [7.0]	0.50 [0.03]	4.4 [5.8]	0.01
** Warm Ischemia Time [min]**	28.5 [5.5]	28.6 [5.5]	31 [5.6]	27.5[6.0]	0.95

**Table 2:** Tubular Injury Scoring Scale.

Tubular Injury Score	Description
**0**	Normal tubules no evidence of injury
**1.0**	Mild focal tubular dilatation with occasional epithelial attenuation
**2.0**	Patchy moderate tubular dilatation, more extensive epithelial attenuation and edema
**3.0**	Moderate to marked tubular dilatation with diffuse tubular attenuation, edema and frank tubular epithelial necrosis

### Immunohistochemistry

Immunohistochemistry was performed on formalin-fixed 5 μm thick paraffin embedded sections. Sections were deparaffinized with xylene followed by rehydration with graded ethanol to water. For immunostaining of the FL-MMP-2, the sections were incubated for 30 minutes with a prediluted monoclonal anti-mouse antibody against the N-terminal sequence of the MMP-2 protein (Abcam ab54401), followed by a 30 minute incubation with biotinylated anti-mouse IgG (Vector). For immunostaining of the NTT-MMP-2 isoform, the sections were incubated overnight at 4°Celsius with 5 μg/ml of a polyclonal goat IgG targeting the catalytic sequence exposed by the absence of the N-terminal propeptide in this isoform. To identify peritubular capillaries, antigen retrieval was performed with Vector antigen unmasking solution and the sections were stained for the endothelial cell marker CD 31 using a prediluted anti-mouse monoclonal antibody (Abcam ab958). After each primary antibody the sections were incubated for 30 minutes with secondary biotinylated antibody (Vector), followed by an incubation with Vectastain ABC complex (Vector) and then immunohistochemical development was performed using VIP peroxidase substrate (Vector). All slides were counterstained with methyl green.

To determine correlations of MMP-2 isoform staining with tubular injury, serial sections of individual biopsies were aligned in the sequence NTT-MMP-2, FL-MMP-2 and hematoxylin/eosin and ranked using the criteria outlined in Tables 1 and 2.

Peritubular capillary density on CD 31-stained biopsies was quantified according to Choi, et al. [21]. Quantitative analyses were performed on 8 control biopsies and 8 DGF biopsies with injury scores of 2–3. In brief, the total number of peritubular capillaries within each of 10 random 0.25 mm^2^ fields viewed at 200X were counted and the density expressed as the mean number of capillaries/field.

### TUNEL staining and quantification

Fragmentation of tubular epithelial cell nuclear DNA was determined with the TACS-XL Blue kit according to the manufacturer’s instructions (R & D Systems). A random subset of 29 biopsies, including 5 controls, were de-identified, stained and the number of TUNEL-positive tubular epithelial cells counted in 10 transversely cut tubules in each biopsy specimen. Subsequent analyses correlated the number of TUNEL-positive tubular epithelial cells as a function of the tubular injury score detailed above and as a function of the level of IHC staining for the two MMP-2 isoforms.

### RNA *in situ* hybridization assay for the NTT-MMP-2 transcript

To perform the RNA in situ hybridization assay the RNAscope 2.0 FFPE assay kit was used (Advanced Cell Diagnostics). Formalin-fixed paraffin embedded sections were mounted on Superfrost Plus slides (Fisher Scientific). The sections were deparaffinized with xylene followed by dehydration with 100% ethanol and endogenous peroxidase blocking using the RNAscope endogenous enzyme blocker. The slides were then boiled in citrate buffer for 15 minutes, rinsed in deionized water and immediately treated with protease at 40°C for 30 minutes. After the protease digestion, the sections were incubated sequentially with RNAscope custom oligonucleotide probes. The oligonucleotide probes were targeted against 500 bp of the first intron of the human MMP-2 gene immediately adjacent to the second exon, a sequence which represents the 5’UTR of the NTT-MMP-2 transcript.

The oligonucleotide probes were hybridized at 40°C for 2 hours. Thereafter, preamplifier at 40°C for 30 minutes, a signal enhancer at 40°C for 15 minutes, amplifier at 40°C for 30 minutes, the label probe at 40°C for 15 minutes, a signal amplifier for 30 minutes at room temperature and lastly sections were incubated with an alkaline phosphatase-linked labeling molecule for 15 minutes at room temperature. After each step, sections were washed two times with the RNAscope 2.0 wash buffer at room temperature. Hybridization signal detection was performed with the RNAscope 2.0 Fast Red reagent diluted to 1:60 for 10 minutes, followed by counterstaining with Gill’s hematoxylin.

### Quantitative reverse transcription polymerase chain reaction (qPCR)

To isolate total RNA, human renal biopsy samples (three 15 μ sections/biopsy) were deparafinized in xylene. RNA was extracted using an RNAeasy FFPE kit (Qiagen) according to the manufacturer’s instructions. Isolated RNA was quantified and normalized to synthesize cDNA using a Quantitect Reverse Transcription Kit (Qiagen). qRT-PCR was performed to quantify the relative expression of FL-MMP-2 and NTT-MMP-2 transcripts between DGF biopsies and controls using a LightCycler 480 SYBR Green I Master kit (Roche Applied Bioscience Inc.). Each clinical sample was plated in triplicate in a 384 well PCR plate (Thermo Fisher). Specific primers were designed and synthesized to assay for the human FL-MMP-2 transcript (F: 5’-TCGCCCATCATCAAGTTCCC-3’, R: 5’-GGGCAGCCATAGAAGGTGTT-3’) and NTT-MMP-2 transcript (F: 5’-TCCTGTCTGGACTATGGCACT-3’, R: 5’-GGCAGCCATAGAAGTGTGTTC-3’). Amplification reactions were performed with 40 cycles (95°C for 15 sec; 58°C for 45 s; and 72°C for 1 min) and normalized to the ribosomal protein 36B4 transcript [22]. Melt curves were used to verify absence of primer dimers and other non-specific products in the amplification reactions. We used the 2 –^ΔCt^ method of analysis since the values are from different patient samples [23].

### Statistical methods

Continuous data were summarized as mean and standard deviation for approximately normally distributed variables and as median and interquartile range for non-normally distributed variables. Categorical variables were summarized using proportions. ANOVA and Chi square analysis were employed for testing of differences in clinical characteristics based on transplant type.

Tubular injury biopsies are described using our scoring classification in Table 1. FL-MMP-2 and NTT-MMP-2 isoform staining characteristics were described categorically (Table 2). We hypothesized that the tubular injury score was associated with FL-MMP-2 and NTT-MMP-2 isoform transcript abundance as quantified by qRT-PCR. Thus, the tubular injury score was treated as the dependent variable while normalized 2 –^ΔCt^ values for both the FL-MMP-2 and NTT-MMP-2 isoforms were treated as independent variables and were modeled using linear regression, separately and together. In addition, we examined the association of the number of TUNEL-positive tubular epithelial cells with the tubular injury score and the IHC staining score using linear regression. The following covariates were included in multivariate analysis: cold ischemia time, warm ischemia time, and expanded-criteria donor (ECD) status. These covariates were based on previous studies demonstrating a potentially causal relationship with DGF associated to ischemia/reperfusion injury [4,24–27]. Differences in transcript abundance and tubular injury were calculated using Spearman Correlation. A p value of less than 0.05 was considered statistically significant. Analyses were performed using STATA version 13 (College Station, Texas, USA).

## Results

### Clinical characteristics

Data from 51 individuals were abstracted and included in the analysis (Table 1). There were 10 controls and 41 cases with a clinical diagnosis of DGF. The mean age of the group was 54 ±12 years, and the majority were male (72.5%). Deceased donor transplant was the most common transplant type (78%). The median dialysis vintage was 48 months (interquartile range [IQR] 21, 72 months). Dialysis vintage was longer for recipients of deceased donor transplant (58 ± 40 months, p = 0.05). The median cold ischemia time was 8 hours ([IQR] 0.5, 12 hours).

### Classification of tubular Injury

**Fig 1:**
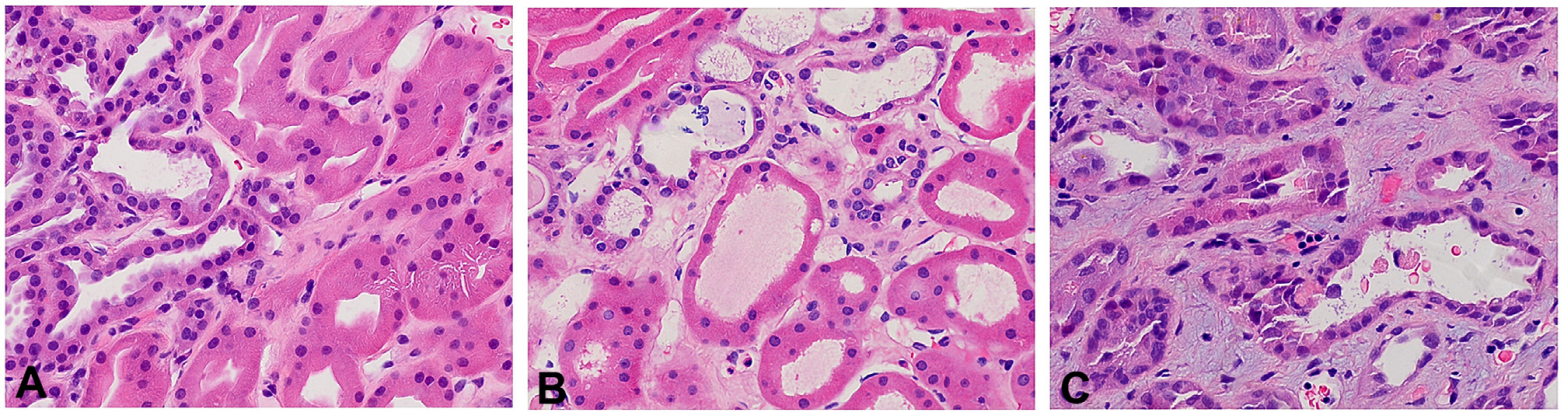
Scoring of injury in H/E stained delayed graft function kidney biopsies. Panel A: Injury score of 1-mild focal tubular dilatation; Panel B: Injury score of 2-biopsy shows more extensive tubular dilatation, mild interstitial edema, and occasional necrotic tubular epithelial cell in lumen; Panel C: Injury score of 3-moderate to severe tubular dilatation, extensive interstitial edema, and frequent necrotic tubular epithelial cells in lumen. (Final magnification X 400).

The scoring criteria for assessing renal tubular injury are outlined in Table 2. Fig 1 provides representative examples of hematoxylin/eosin-stained transplant biopsies with tubular injury scores ranging from 1 to 3. It is important to note that the entire renal biopsy core was used to determine the extent of tubular injury. Tubular injury classification was completed for 48 of the 51 cases. Eleven biopsies were classified as not having evidence of tubular injury (injury score: 0). Seven cases were classified with mild focal dilatation with occasional epithelial cell loss (injury score: 1). Fifteen biopsies were scored with patchy moderate tubular dilatation with edema and some epithelial cell necrosis (injury score: 2). Fifteen biopsies were classified as the most severe with moderate to marked tubular dilatation with diffuse tubular attenuation, edema and frank tubular epithelial necrosis (injury score: 3). Stratified by transplant type, the mean tubular injury score for deceased donors was 1.97 ±1.0; for living unrelated donors 1.66 ± 0.60; and living related donors 0.50 ±.75 (p = 0.003).

### Delineation of tubular immunohistochemistry scores

**Fig 2:**
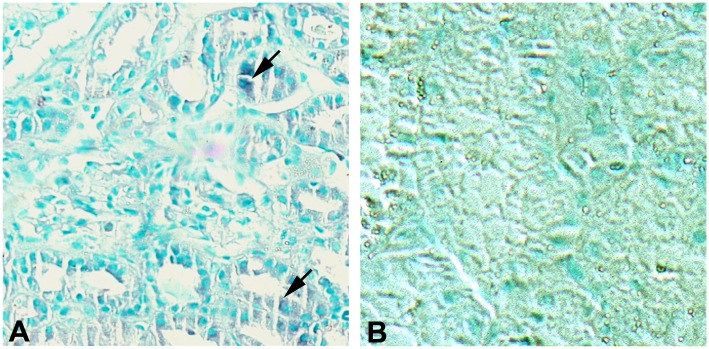
FL-MMP-2 and NTT-MMP-2 expression in control protocol renal biopsies. Panel A: Control protocol biopsy stained for FL-MMP-2. There is trace, focal immunohistochemical staining for FL-MMP-2 in proximal tubules (arrows) Panel B: Control protocol biopsy stained for NTT-MMP-2. There is no detectable immunohistochemical staining. (Final magnification X 200).

The immunohistochemistry staining scores for FL-MMP-2 and NTT-MMP-2 are provided in Table 3. Fig 2 demonstrates the immunohistochemical results achieved with staining normal control protocol renal biopsies for FL-MMP-2 and NTT-MMP-2. There is trace proximal tubular epithelial cell staining for FL-MMP-2 in the controls; there was no detectable NTT-MMP-2 staining in any of the controls.

**Table 3:** Immunohistochemical staining score system for FL- & NTT-MMP-2 isoforms in renal biopsies.

Stain Score	Description
**0**	Negative staining
**1.0**	Patchy staining
**2.0**	Moderate diffuse staining
**3.0**	Dense diffuse staining

MMP-2 and NTT-MMP-2 isoform staining results was available for 48 individuals. The largest proportion of cases for FL-MMP-2 staining were classified as moderate diffuse (score 2.0) and dense diffuse (score 3.0). For the NTT-MMP-2 isoform the most commonly allocated score was moderate diffuse (score 2.0). Deceased donor transplants had a trend towards a higher staining score for both the MMP2 and NTT-MMP-2 isoforms, although this was not statistically significant.

### Tubular epithelial cell expression of FL-MMP-2 and NTT-MMP-2 is directly associated with tubular injury

**Fig 3:**
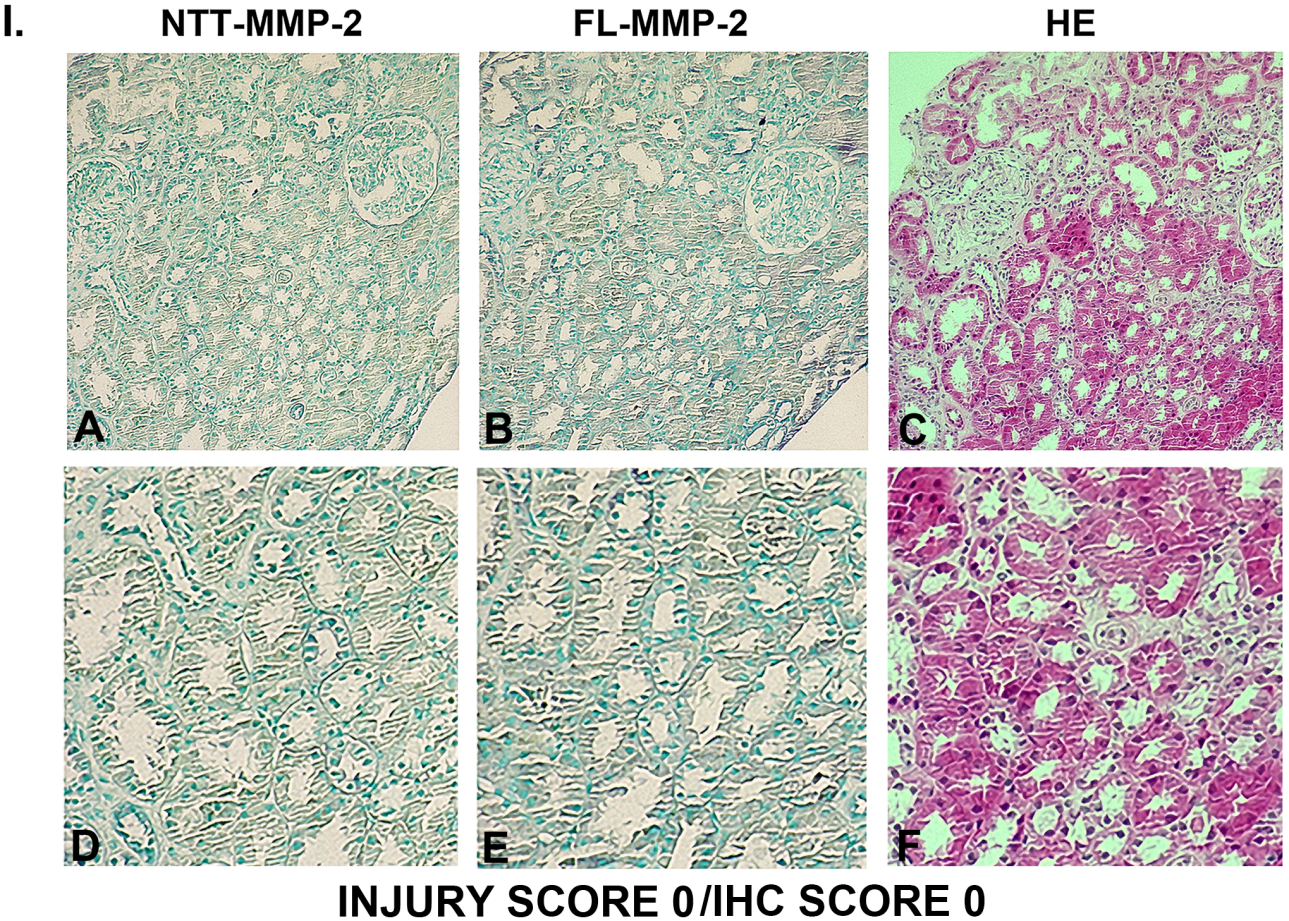
Ranking of serial biopsy sections for degree of tubular injury and immunohistochemistry staining. Serial sections of protocol and DGF biopsies were stained for NTT-MMP-2 and FL-MMP-2 with hematoxylin/eosin and ranked according to injury score. Representative results for injury score 0. (A, B, C X 300; D, E, F X 600).

**Fig 4:**
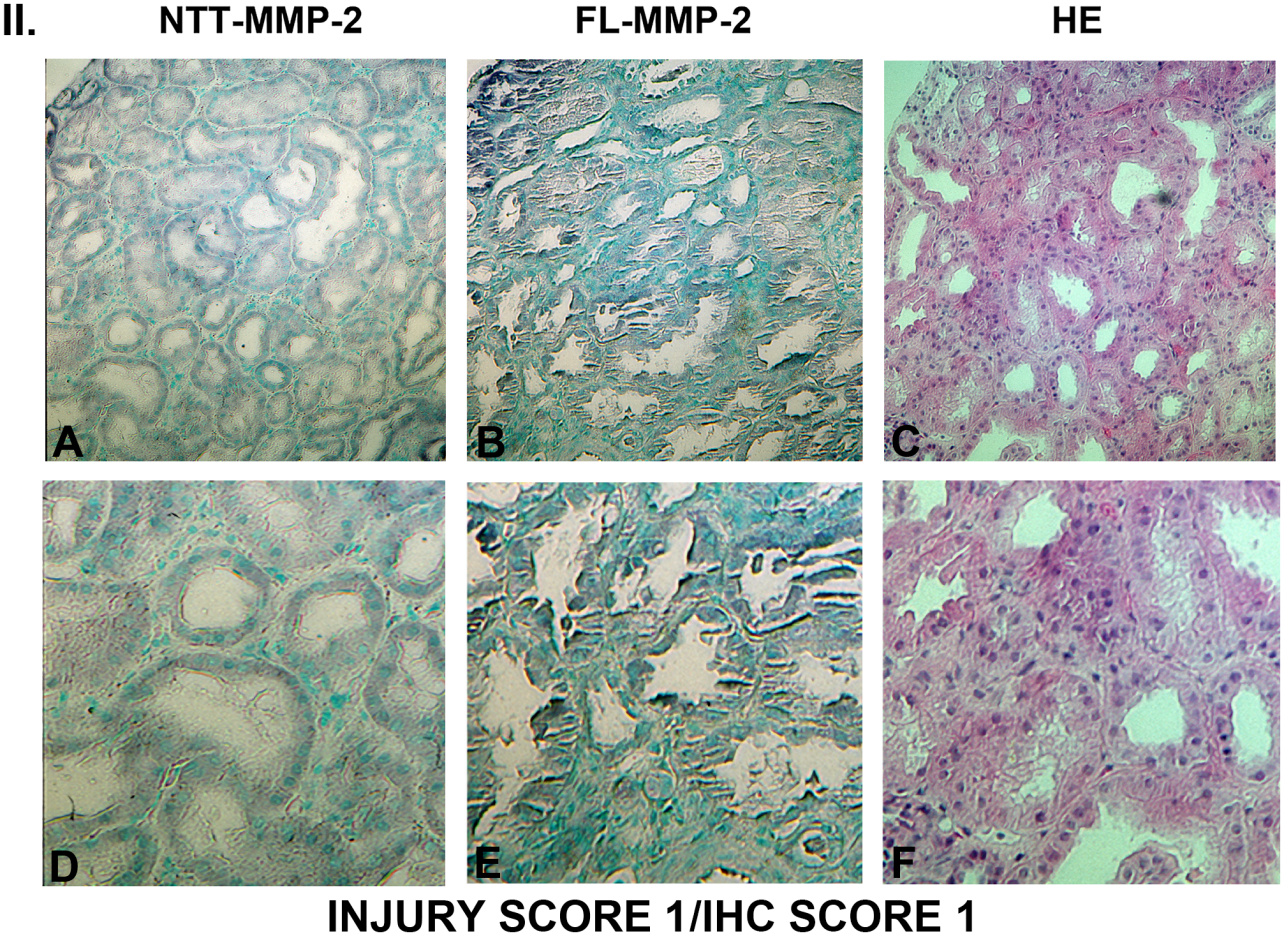
Ranking of serial biopsy sections for degree of tubular injury and immunohistochemistry staining. Serial sections of protocol and DGF biopsies were stained for NTT-MMP-2 and FL-MMP-2 with hematoxylin/eosin and ranked according to injury score. Representative results for injury score 1. (A, B, C X 300; D, E, F X 600).

**Fig 5:**
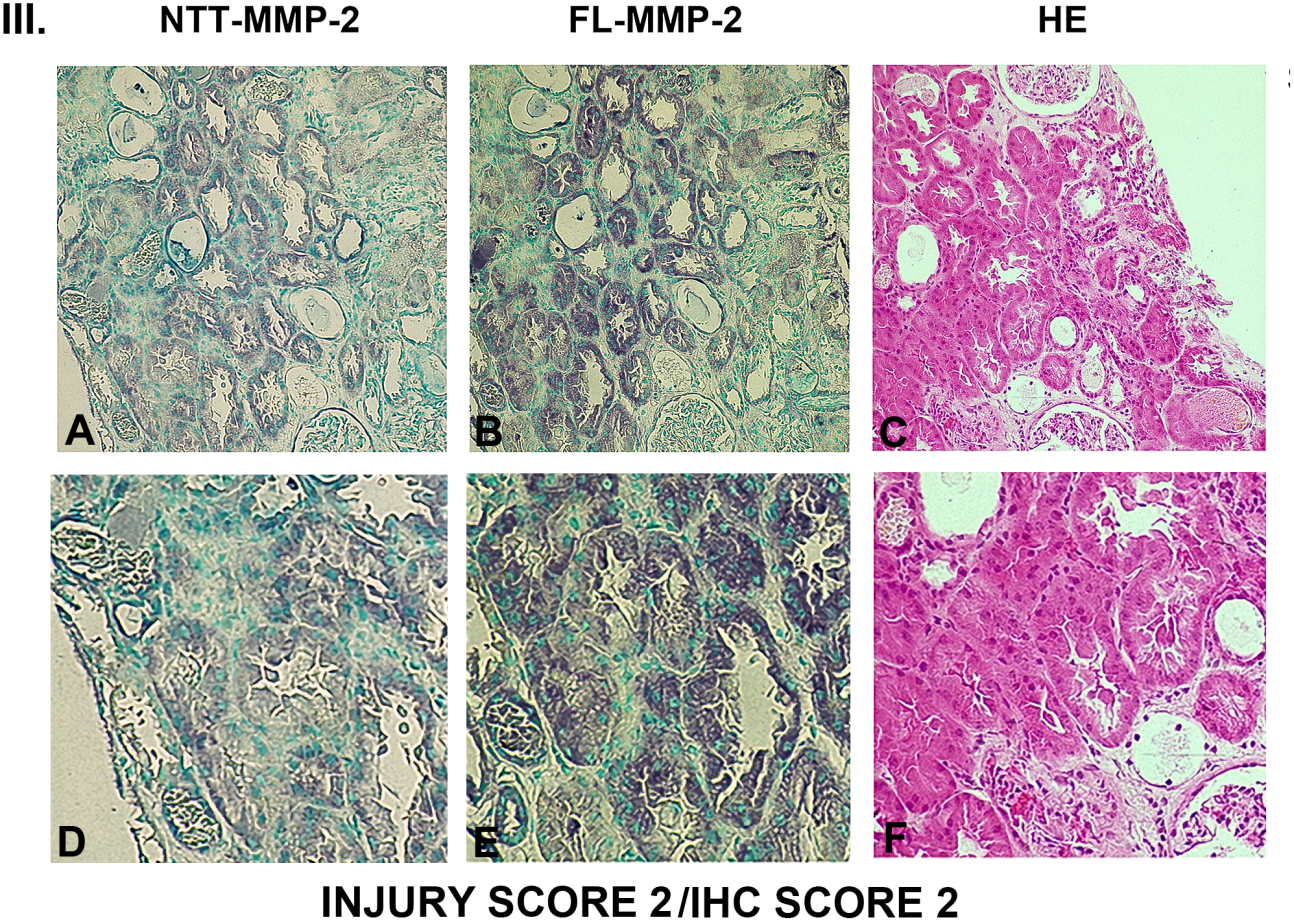
Ranking of serial biopsy sections for degree of tubular injury and immunohistochemistry staining. Serial sections of protocol and DGF biopsies were stained for NTT-MMP-2 and FL-MMP-2 with hematoxylin/eosin and ranked according to injury score. Representative results for injury score 2. (A, B, C X 300; D, E, F X 600).

**Fig 6:**
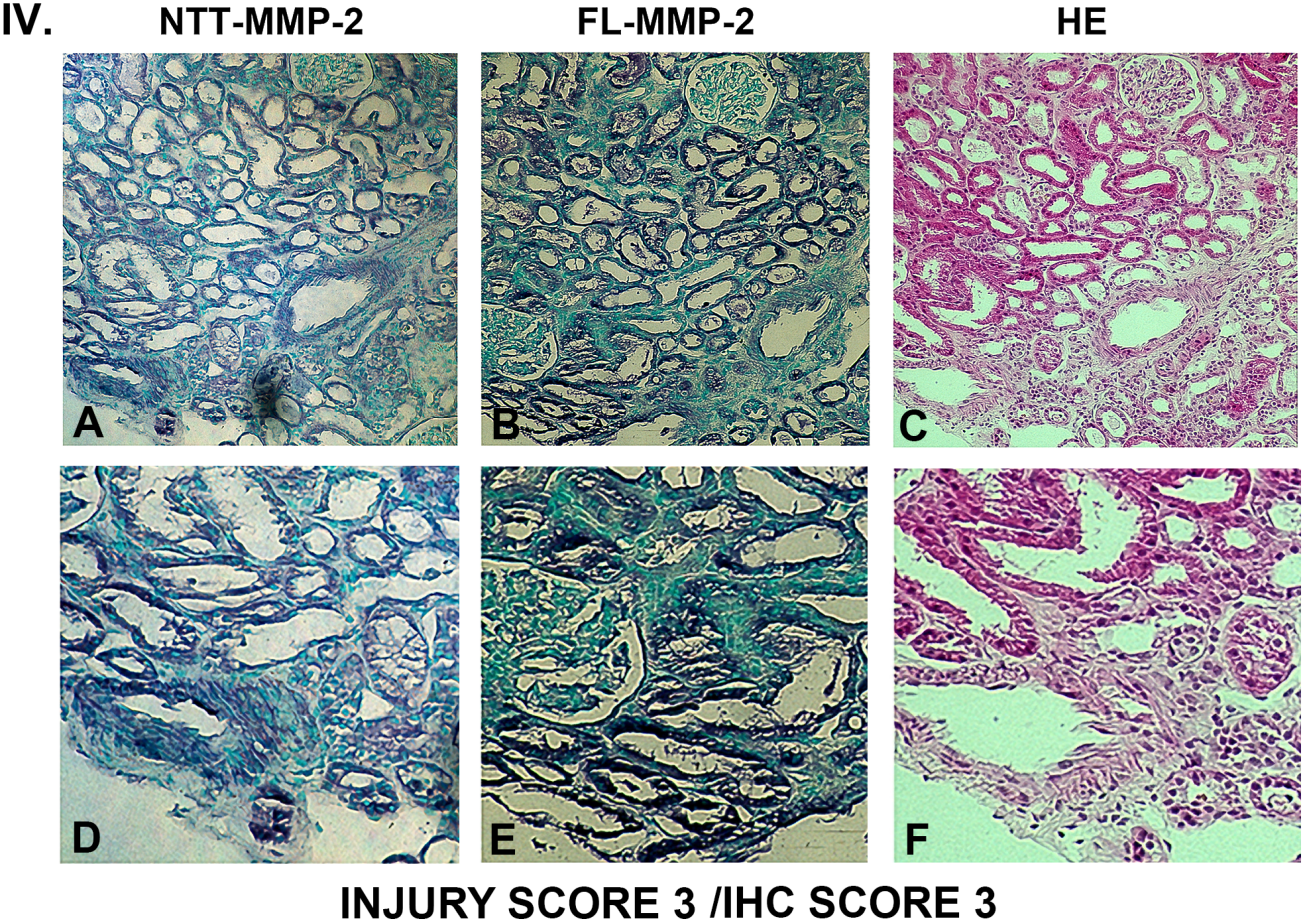
Ranking of serial biopsy sections for degree of tubular injury and immunohistochemistry staining. Serial sections of protocol and DGF biopsies were stained for NTT-MMP-2 and FL-MMP-2 with hematoxylin/eosin and ranked according to injury score. Representative results for injury score 3. (A, B, C X 300; D, E, F X 600). There is a correlation between the overall tubular injury scale and the immunohistochemical staining scales for both NTT-MMP-2 and FL-MMP-2. Significantly, immunohistochemical staining for both NTT-MMP-2 and FL-MMP-2 occurs within the same tubular segments and there is a direct correlation between NTT-MMP-2 and FL-MMP-2 staining tubular injury.

Figs 3, 4, 5 and 6 are comprised of serial sections of DGF renal biopsies ranked according to immunohistochemical staining intensity and tubular injury score. Grading reflects analyses of the entire biopsy core. Note that the staining intensity for FL-MMP-2 is greater than NTT-MMP-2 for all levels of tubular injury. This is consistent with the results of qPCR detailed below, in which the transcript abundance of NTT-MMP-2 is considerably less than that of the FL-MMP-2 isoform. The serial sections demonstrate that staining for the FL-MMP-2 and NTT-MMP-2 isoforms occurs within the same tubular cellular segments. Most importantly, the serial sections demonstrate that the tubular segments with the most prominent FL and NTT-MMP-2 isoform expression align with the areas of tubular epithelial cell injury in the H/E-stained serial sections Thus, FL-MMP-2 and NTT-MMP-2 are co-expressed and tubular injury is linked to the location and intensity of expression of both MMP-2 isoforms.

### Association of TUNEL tubular epithelial cell staining with tubular injury score and MMP-2 isoform IHC score

TUNEL staining was used to quantify nuclear DNA fragmentation of tubular epithelial cells as detailed in Materials and Methods. The numbers of TUNEL-positive tubular epithelial cells were correlated to the tubular injury score. TUNEL-positive tubular epithelial cells demonstrated a highly significant correlation with the tubular injury scoring scale. Tubular injury scores of 0, 1, 2 and 3 were associated with 3.0±2.0; 12.2±3.2; 29.0±5.4 and 61.5±19 TUNEL-positive tubular epithelial cells, respectively (n = 6-7/group, p<0.01). Control biopsies contained 0.4±0.5 TUNEL-positive cells (n = 5). TUNEL-positive cells also demonstrated a correlation with the IHC staining score for both MMP-2 isoforms. A single unit increase in FL-MMP-2 staining score was associated with the presence of a significantly higher number of TUNEL-positive tubular epithelial cells (ρ = 18.81; p<0.01). Further, a single unit increase in NTT-MMP-2 staining score was also highly correlated with increased TUNEL-positive tubular epithelial cells (ρ = 19.43; p<0.01).

### FL-MMP-2 and NTT-MMP-2 tubular epithelial cell staining are in distinct intracellular locations

**Fig 7:**
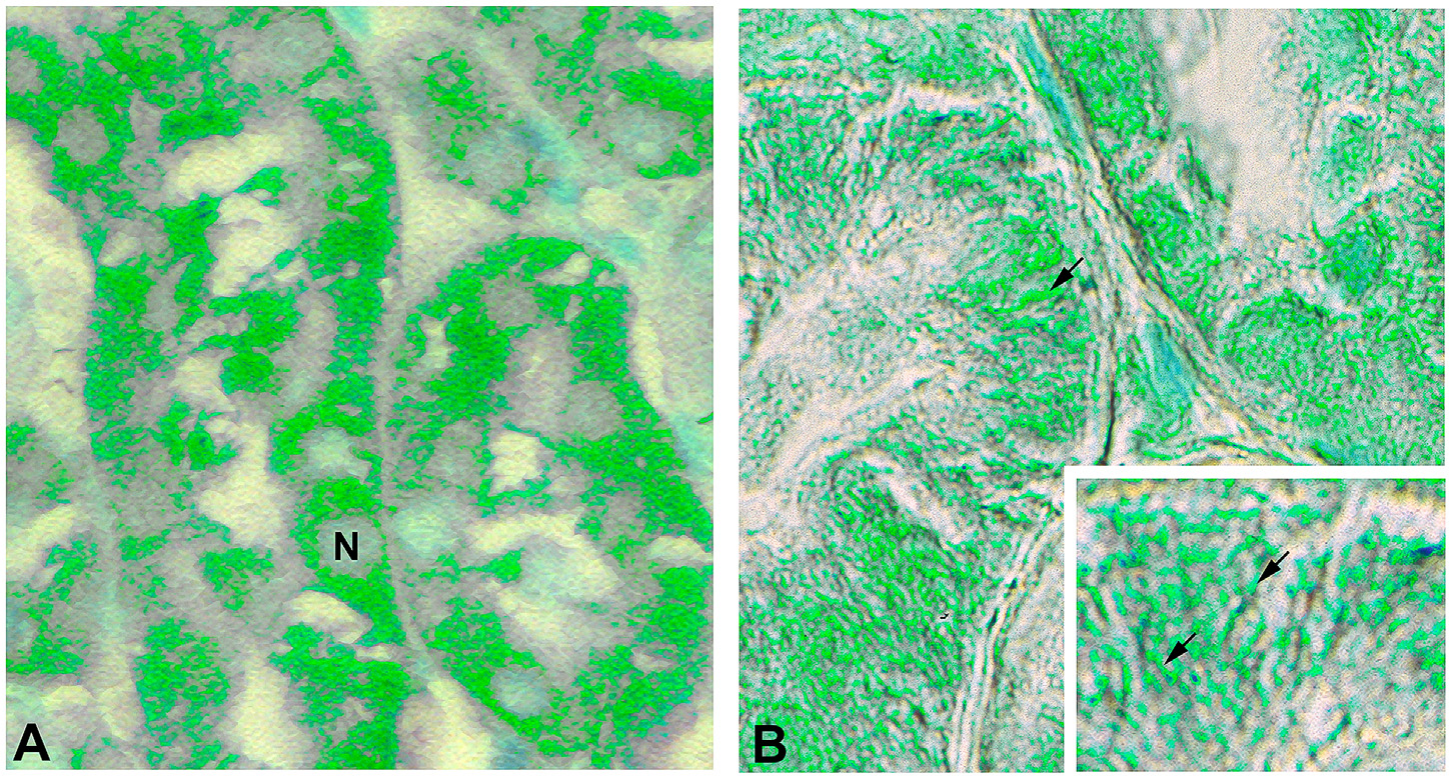
FL-MMP-2 and NTT-MMP-2 are found within discrete tubular epithelial cellular compartments. Images of DGF biopsies were acquired using Nomarksi optics and processed with pseudocolor to enhance contrast as detailed in Materials and Methods. Panel A: Immunohistochemical staining for FL-MMP-2 is confined to the cytoplasmic compartment (N, nucleus). Panel B: Immunohistochemical staining for NTT-MMP-2 is concentrated at the basolateral surfaces of the proximal tubular epithelial cells adjacent to the tubular basement membrane. The insert demonstrates NTT-MMP-2 staining within extended filamentous structures contained within basolateral infoldings characteristic of mitochondria. (Final magnification Panels A, B: X600, insert X1200).

The antibody used to detect the NTT-MMP-2 isoform is directed against an epitope located in the catalytic domain of the enzyme. The catalytic domain of FL-MMP-2 is not accessible to the NTT-MMP-2 antibody due to steric hindrance from the overlying N-terminal prodomain. It was theoretically possible; however, that the NTT-MMP-2 antibody could detect active FL-MMP-2 in which the N-terminal prodomain was released by oxidative stress. In this setting the N-terminal prodomain sequence would not limit NTT-MMP-2 antibody access to the catalytic domain epitope. We have previously reported that small amounts of the FL-MMP-2 isoform are present within highly purified cytosolic fractions, while the NTT-MMP-2 isoform was present within the intramembranous space of mitochondria [20]. To address this issue we obtained high resolution Nomarski optics images of DGF sections stained with either the FL-MMP-2 or NTT-MMP-2 antibodies (Fig 7). The Nomarski images are displayed as pseudocolor images to enhance contrast. Using the anti-FL-MMP-2 antibody, immunohistochemical signal is diffusely present within the tubular epithelial cells, consistent with a cytosolic localization (Panel A). In contrast, signal obtained with the NTT-MMP-2 antibody is concentrated within the basolateral aspects of the tubular epithelial cells in long filamentous structures characteristic of mitochondria. There was no meaningful overlap of the respective immunohistochemical signals, confirming the selective affinities of the antibodies employed.

### *In situ* hybridization confirms the expression of the NTT-MMP-2 isoform in DGF kidneys

**Fig 8:**
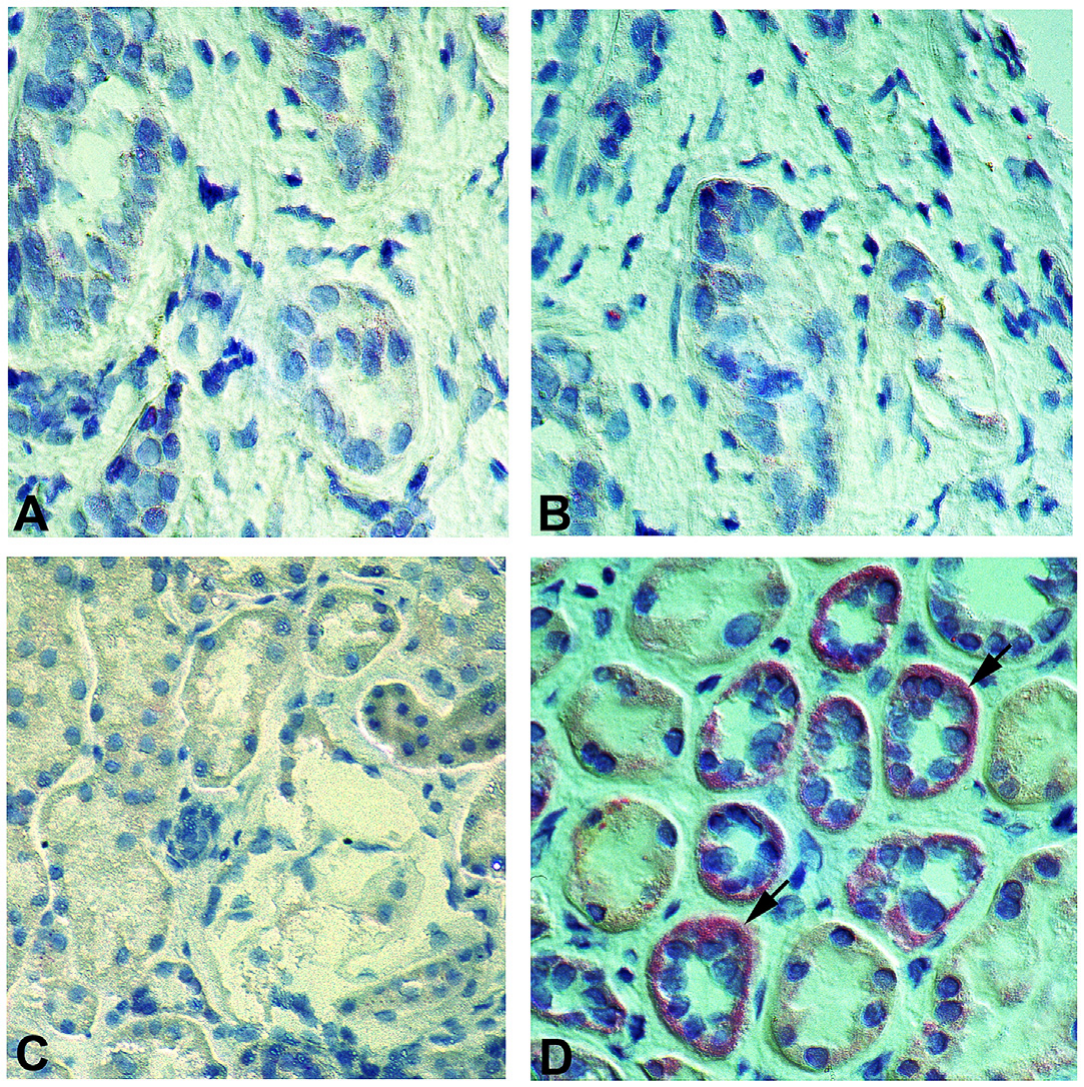
*In situ* hybridization confirms NTT-MMP-2 expression in DGF tubular epithelial cells. Control protocol biopsies and DGF biopsies were processed for in situ hybridization to detect the NTT-MMP-2 mRNA transcript as detailed in Materials and Methods. Panel A: Control renal biopsy without the targeting NTT-MMP-2 oligonucleotides. Panel B: Control renal biopsy including the targeting NTT-MMP-2 oligonucleotides. There is no detectable NTT-MMP-2 transcript. Panel C: DGF biopsy without the targeting NTT-MMP-2 oligonucleotides. Panel D: DGF biopsy with the targeting NTT-MMP-2 oligonucleotides. There is readily detectable NTT-MMP-2 mRNA transcript signal within tubular epithelial cells (arrows). (Final magnification X 300).

We performed in situ hybridization for the NTT-MMP-2 transcript in order to confirm our results with immunohistochemistry. The NTT-MMP-2 isoform is generated by oxidative stress-mediated activation of an alternative promoter located in the 3’ region of the first intron of the MMP-2 gene. Transcription from this alternative promoter generates a unique 500 bp 5’ UTR that is spliced out of the pre-mRNA of the FL-MMP-2 isoform. The 5’ UTR of NTT-MMP-2 served as the target template for the in situ hybridization studies. Representative results are depicted in Fig 8. Panel A is a control renal biopsy in situ hybridization lacking the targeting oligonucleotides, while Panel B is a control renal biopsy in situ hybridization that included the targeting oligonucleotides. No appreciable hybridization signal is seen in either sample. Panel C is a DGF biopsy in situ hybridization that lacks the targeting oligonucleotides, while Panel D is a DGF biopsy including the oligonucleotides. Prominent hybridization signal (red) is seen within the cytoplasm of the tubular epithelial cells in Panel D, thereby confirming that the NTT-MMP-2 isoform is indeed present within DGF biopsies.

### Quantitative PCR of FL-MMP-2 and NTT-MMP-2 transcripts in renal biopsies confirms up-regulation of both transcripts

**Fig 9:**
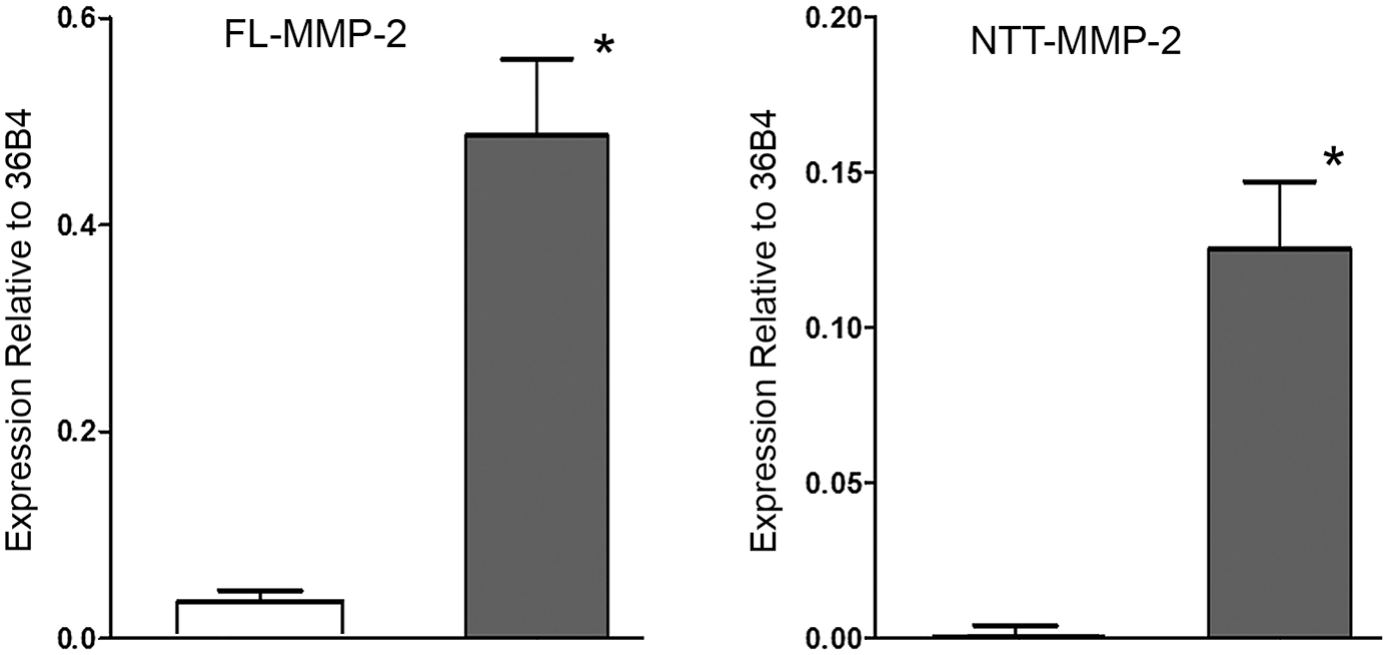
qPCR measurement of FL-MMP-2 and NTT-MMP-2 transcript abundance in control protocol biopsies and DGF biopsies. mRNA was extracted from paraffin-embedded formalin-fixed renal biopsy specimens and FL-MMP-2 and NTT-MMP-2 transcript abundance was determined by qPCR as detailed in Materials and Methods. Both isoforms were normalized to a ribosomal protein, 36B4. There was a low, but detectable level of FL-MMP-2 transcript in the control protocol biopsies, consistent with the level of FL-MMP-2 immunohistochemical staining shown in Fig 2. FL-MMP-2 transcript abundance was increased approximately twelve-fold in the DGF samples. NTT-MMP-2 transcript abundance was nearly undetectable in the control protocol biopsies, consistent with the absence of NTT-MMP-2 immunohistochemical staining in controls (Fig 2). In contrast, NTT-MMP-2 transcripts were readily detectable in the DGF biopsies (* p<0.01).

We were able to recover sufficient template RNA from the formalin-fixed, paraffin embedded biopsy specimens to perform qPCR for the MMP-2 isoforms. The results of these analyses are presented in Fig 9. Relative to the normalizing ribosomal 36B4 transcript, expression of the FL-MMP-2 transcript in the controls was 0.04±03 and 0.49±0.10 in the DGF group (p<0.01), which represents an approximate ten-fold increase in FL-MMP-2 transcript abundance in the setting of DGF. NTT-MMP-2 transcripts were at the limits of detection in the controls (0.0004±0001 relative to 36B4), but were greatly increased in the DGF samples (0.125±.001 relative to 36B4, p<0.001). The ratio of the FL-MMP-2 and NTT-MMP-2 transcript abundance is approximately 4:1, which reasonably correlates with the relative expression levels obtained with immunohistochemistry. Further, the qPCR results provide independent molecular validation for the results obtained by immunohistochemistry and in situ hybridization.

### DGF is associated with peritubular capillary rarefaction

**Fig 10:**
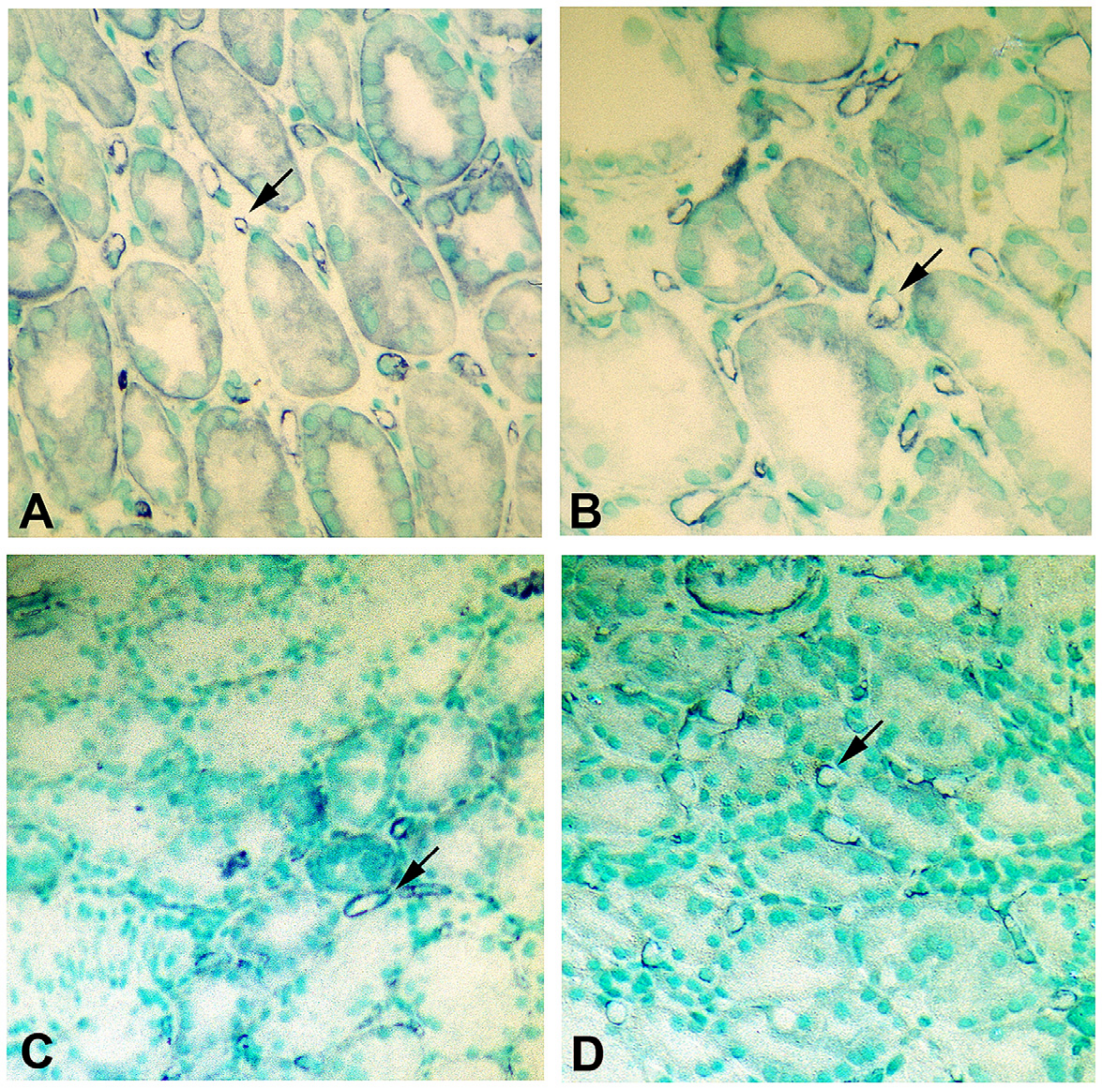
Delayed graft function is associated with decreased peritubular capillary density. Capillary endothelial cells were detected by CD-31 staining as detailed in Materials and Methods. Panel A, B: CD-31 staining of control protocol biopsy. Tubular structures are surrounded by multiple peritubular capillaries (arrows). Panel C, D: CD-31 staining of DGF biopsy. There is an evident decrease in the abundance and density of the peritubular capillaries, particularly in a patchy, non-uniform distribution (arrows). (Final magnifications: Panels A, C: X250; Panels B, D: X 300).

We used CD31 immunohistochemistry to detect peritubular capillary endothelium in control and DGF biopsies. Representative results are depicted in Fig 10. In the controls there is a relatively dense network of peritubular capillaries surrounding the individual nephron segments, while in the DGF biopsies there was extensive drop out in a patchy distribution of the peritubular capillaries. Quantitative assessment of peritubular capillary density in the controls demonstrated 26.9±3.3 capillaries/0.25 mm^2^ field. Peritubular capillary density in the DGF group was significantly reduced to 15.9±32 capillaries/0.25 mm^2^ field; p<0.01.

### Statistical associations of FL-MMP-2 and NTT-MMP-2 transcript abundance with tubular injury

Differences in transcript abundance were correlated with tubular injury score (FL-MMP-2, ρ = 0.36, p = 0.03); (NTT-MMP-2, ρ = 0.49, p = 0.002). In unadjusted linear regression analysis, higher abundance of FL-MMP-2 transcripts was associated with higher tubular injury score (1.10 points higher inflammation score per unit increase of FL-MMP-2 transcript abundance, 95%CI 0.17, 2.03; Table 4). Adjusting for cold ischemia time did not significantly alter the association of FL- MMP-2 transcript abundance with tubular injury score, (1.25 points higher inflammation score per unit increase of FL-MMP-2 transcripts abundance, 95%CI 0.18, 2.32). Cold ischemia time was not independently associated with tubular injury score when included in the model (p>0.05). Higher concentrations of FL-MMP-2 transcripts were associated with higher tubular injury score when adjusted for ECD transplant status (1.10 points higher inflammation score per unit increase of FL-MMP-2 transcripts abundance, 95%CI 0.16, 2.05). Higher concentrations of FL-MMP-2 transcripts remained associated with higher tubular injury score after adjusting for both cold ischemia time and ECD transplant status (1.21 points higher inflammation score per unit increase of FL-MMP-2 transcript abundance, 95%CI 0.11, 2.30).

**Table 4:** Linear regression modeling of the association of FL-MMP-2 isoform transcript abundance with tubular injury.

Variables	Unadjusted Coef [95%CI]	Adjusted for Cold Ischemia Time Coef [95%CI]	Adjusted for Extended Donor Criteria Transplant Coef [95%CI]	Adjusted for Cold Ischemia Time and Extended Donor Criteria Transplant Coef [95%CI]
**FL-MMP-2**	1.10 [0.17, 2.03]	1.25 [0.18, 2.32]	1.10 [0.16, 2.05]	1.21 [0.11,2.30]
**Cold Ischemia, hr**	--	-0.007 [-0.06, 0.05]	--	-0.002 [-0.06,0.06]
**ECD**	--	--	0.25 [-0.94, 1.45]	0.30 [-0.99,1.58]

As compared to FL-MMP-2, there was a stronger unadjusted association between NTT-MMP-2 transcript abundance and tubular injury score (Table 5). Higher concentrations of NTT-MMP-2 transcripts were associated with higher tubular injury score (3.42 points higher inflammation score per unit increase of NTT-MMP-2 transcripts abundance, 95%CI 0.24, 6.61). Cold ischemia time was not associated with tubular injury score when included in the model (p>0.05). However, the association of NTT-MMP-2 transcript abundance with tubular injury score was slightly higher (3.82 points higher inflammation score per unit increase of NTT-MMP-2 transcript abundance, 95%CI 0.22, 7.43). When ECD status was included in the model, the association of NTT-MMP-2 transcript abundance with tubular injury score remained statistically significant (3.44 points higher inflammation score per unit increase of NTT-MMP-2 transcripts abundance, 95%CI 0.21, 6.67). Higher concentrations of NTT-MMP-2 transcripts with higher tubular injury score after adjusting for both cold ischemia time and ECD transplant status did not reach statistical significance (3.69 points higher inflammation score per unit increase of FL-MMP-2 transcript abundance, 95%CI -0.0008, 7.38).

**Table 5:** Linear regression modeling of the association of NTT-MMP-2 transcript abundance with tubular injury.

Variables	Unadjusted Coef [95%CI]	Adjusted for Cold Ischemia Time Coef [95%CI]	Adjusted for Extended Donor Criteria Transplant Coef [95%CI]	Adjusted for Cold Ischemia Time and Extended Donor Criteria Transplant Coef [95%CI]
**NTT-MMP-2**	3.42 [0.24, 6.61]	3.82 [0.22, 7.43]	3.44 [0.21, 6.67]	3.69 [-0.0008, 7.38]
**Cold Ischemia, hr**	--	-0.002 [-0.06, 0.06]	--	0.004 [-0.06, 0.07]
**ECD**	--	--	0.25 [-0.96, 1.46]	0.33 [-0.97,1.63]

## Discussion

### Expression of the FL-MMP-2 and NTT-MMP-2 isoforms is directly associated with tubular epithelial cell injury

The goals of our study, which should be considered a pilot project, were to determine if two defined MMP-2 isoforms are induced within the setting of clinical renal transplant DGF. Further, we wished to determine if MMP-2 isoform expression could be quantitatively linked with tubular injury as the primary outcome. The principal findings of our study are that both FL-MMP-2 and NTT-MMP-2 are induced, primarily within proximal tubules, in the setting of DGF. In addition, we show that FL-MMP-2 and NTT-MMP-2 are co-induced in the same tubular epithelial cells and that epithelial cell expression is directly associated with histologic evidence of cellular injury and death. We note that there was a strong association between the number of TUNEL-positive tubular epithelial cells, the tubular injury score and, perhaps most significantly, the degree of MMP-2 isoform IHC staining.

Statistically significant associations between the extent of FL-MMP-2 and NTT-MMP-2 transcript abundance and morphologic tubular injury were shown, with a higher association for the NTT-MMP-2 isoform. Transcript abundance of the FL-MMP-2 isoform was statistically associated with tubular injury even after adjustment for ECD and cold ischemia time. The transcript abundance of NTT-MMP-2 isoform was statistically associated with tubular injury after adjustment for either cold ischemia time or ECD. We used a complementary panel of techniques, including in situ hybridization and qPCR to confirm the impressions obtained with immunohistochemistry alone. Additional pathologic effects of MMP-2 expression were confirmed by finding a loss of peritubular capillaries, an activity previously ascribed to MMP-2 in rodent models of renal ischemia/reperfusion injury [13–15].

### Ischemia/reperfusion oxidative stress drives coordinated expression of the FL-MMP-2 and NTT-MMP-2 isoforms

One of the most notable findings of the present study relates to the tightly coordinated co-expression of the FL-MMP-2 and NTT-MMP-2 isoforms within the same tubular epithelial cell. Our prior extensive analyses of FL-MMP-2 transcription identified a relatively weak proximal promoter that drives low level, constitutive transcription [28–30]. This proximal promoter is functional in a broad variety of cells and presumably accounts for the low, but clearly detectable, levels of FL-MMP-2 protein and transcripts observed in the control protocol biopsies. In contrast, NTT-MMP-2 protein was not detected in the control biopsies and the qPCR results were at the limits of transcript detection.

### Enhanced MMP-2 isoform expression affects multiple targets to constitute the DGF phenotype

Tubular epithelial cell injury within the setting of acute ischemia/reperfusion injury was previously primarily ascribed to epithelial cell apoptosis [31]. More recently there has developed an appreciation that tubular epithelial injury in this setting represents a combination of apoptosis and regulated necrosis, the latter being the most significant. We note that DNA fragmentation (and positive TUNEL staining) occurs in both conditions. As opposed to random cellular necrosis, there is growing evidence that tubular epithelial cell necrosis, per se, can be regulated by discrete signaling cascades. These include receptor-interacting protein kinase-dependent regulated necrosis which can be blocked with the kinase inhibitor, necrostatin, leading to partial protection against ischemia/reperfusion injury [31,32].

A second pathway leading to regulated necrosis involves mitochondrial injury with opening of the mitochondrial permeability transition (MPT) [32,33]. We have reported that the NTT-MMP-2 isoform is physically located within the intramembranous space of mitochondria [20]. Cardiac transgenic expression of the NTT-MMP-2 isoform was associated with clear transmission electron microscopic evidence for the mitochondrial permeability transition, including pronounced swelling, loss of organized cristae structure and mitophagy [34]. NTT-MMP-2 proteolytic activity within this critical mitochondrial space could have profound effects on mitochondrial function. Notably, Lange and colleagues have recently characterized the large MMP-2 degradome, which consists of greater than 200 identified substrates, many of which are intracellular in nature [35]. Specifically, this list contains at least eight proteins essential for mitochondrial structure and function, including mitochondrial fission 1 protein, ATP citrate synthase, cytochrome C oxidase and ATP synthase. FL-MMP-2 activity is also regulated by post-transcriptional events, the most relevant of which within the context of DGF is oxidative stress. As originally defined by Schulz and colleagues, oxidative stress disrupts the cysteine switch mechanism whereby the N-terminal prodomain is opened and exposes the catalytically active site of the enzyme [36]. In the current study we identified the presence of intracellular FL-MMP-2 within the cytosol of tubular epithelial cells at the protein level. Due to the type of fixation used for standard renal biopsy processing, we were not able to perform in situ zymography and determine whether this intracellular FL-MMP-2 was enzymatically active. Thus, it is conceivable that additional intracellular targets of MMP-2 contribute to the ultimate DGF tubular epithelial cell phenotype.

We noted evident peritubular capillary rarefaction. Peritubular capillary rarefaction, with resultant tubular epithelial cell hypoxia and chronic redox stress, has long term consequences for tubular epithelial cell function and contributes to the development of tubular atrophy and interstitial fibrosis [37,38]. Further, it is conceivable that chronic tubular epithelial cell redox stress leads to sustained activation of the alternative NTT-MMP-2 intronic promoter which could further contribute to loss of nephron mass.

### Clinical implications of MMP-2 isoform expression in human DGF

To the best of our knowledge, the current study represents the first detailed analysis of the potential role(s) of MMP-2 within the setting of human renal DGF. As such, many of the observations obtained with this defined human model of renal ischemia/reperfusion injury may also apply to the much larger clinical challenge of acute kidney injury in the non-transplant setting. While prior studies using rodent models of renal ischemia/reperfusion injury have suggested MMP-2 as a potential therapeutic target, demonstration of MMP-2 isoform association with tubular injury within the context of human disease represents a significant step forward. It is probable that the final DGF phenotype is a consequence of the combined actions of the FL-MMP-2 and NTT-MMP-2 isoforms against discrete cellular or extracellular substrates located in defined cellular compartments. Thus, it will be important to perform additional studies using selective targeting of the individual isoforms to determine the relative importance of each isoform to the final DGF phenotype. Based on our prior and current studies, and the strength of the association of the NTT-MMP-2 isoform with tubular injury, we hypothesize that this isoform may be the ultimate therapeutic target. Future experimental studies will provide critical insights into which MMP-2 isoform represents the most attractive therapeutic goal.

## Supporting Information

S1 Fig The cDNA for the N-terminal truncated MMP-2 isoform was expressed in murine kidney using the proximal tubular epithelial cell-specific Type I γ-GT promoter. Panel A: Control toluidine blue stained semi-thin section showing normal proximal tubular epithelial structure. Panel B: NTT-MMP-2 transgenic mice showing foci of individual tubular epithelial cells undergoing regulated necrosis (arrows). Note the dilated cellular structures with loss of organized nuclei and organelles, which is characteristic of regulated necrosis. (Final mag X 450).
(PDF)
