# Supplementary material for: Two Distinct Isoforms of Matrix Metalloproteinase-2 Are Associated with Human Delayed Kidney Graft Function
Source: PLoS One. 2015 Sep 17;10(9):e0136276. doi: 10.1371/journal.pone.0136276 (PMC4574928; doi:10.1371/journal.pone.0136276)
Supplement: S1 Fig — Panel A: Control toluidine blue stained semi-thin section showing normal proximal tubular epithelial structure. Panel B: NTT-MMP-2 transgenic mice showing foci of individual tubular epithelial cells undergoing regulated necrosis (arrows). Note the dilated cellular structures with loss of organized nuclei and organelles, which is characteristic of regulated necrosis. (Final mag X 450). (PDF) [file pone.0136276.s001.pdf]

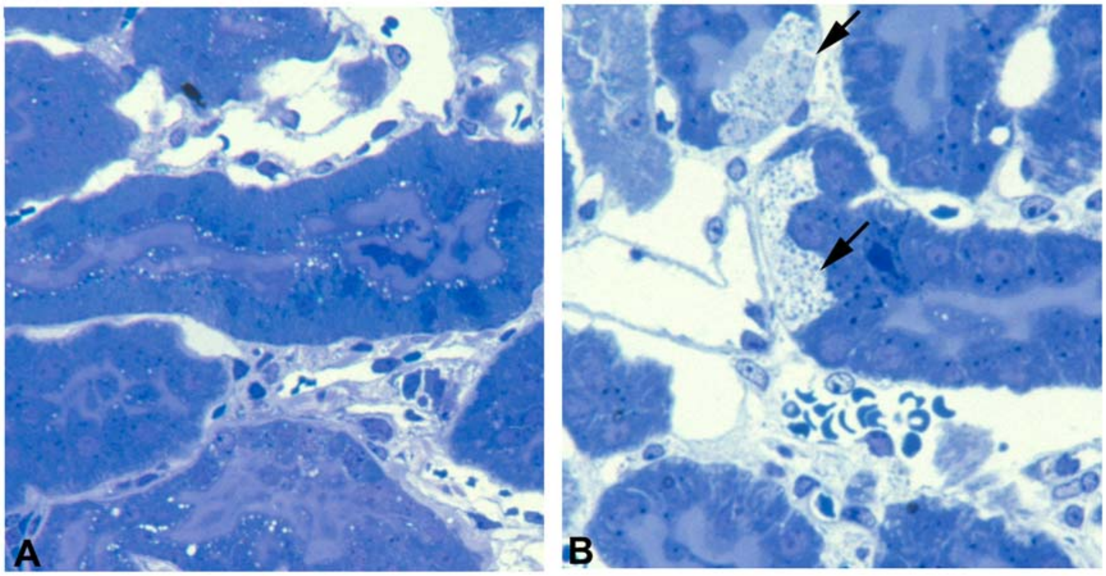

Supplemental Figure 1: The cDNA for the N-terminal truncated MMP-2 isoform was expressed in murine kidney using the proximal tubular epithelial cell specific Type I γ-GT promoter, Panel A: Control toluidine blue stained semi-thin section showing normal proximal tubular epithelial structure.
Panel B: NTT-MMP-2 transgenic mice showing foci of individual tubular epithelial cells undergoing regulated necrosis (arrows). Note the dilated cellular structures with loss of organized nuclei and organelles, which is is characteristic of regulated necrosis. (Final mag X 450)
